# Effects of a 12-Week Municipal Dementia Prevention Program on Cognitive/Motor Functions among the Community-Dwelling Elderly

**DOI:** 10.3390/geriatrics1030018

**Published:** 2016-07-18

**Authors:** Tadahiko Kamegaya, Haruyasu Yamaguchi, Long-Term-Care Prevention Team of Isesaki City Community General Support Center

**Affiliations:** 1 Department of Rehabilitation Sciences, Graduate School of Health Sciences, Gunma University, 3-39-22 Showa-machi, Maebashi-shi, Gunma 371-8514, Japan; yamaguti@gunma-u.ac.jp; 2 Isesaki City Office, 2-410 Imaizumi-cho, Isesaki, Gunma 372-8501, Japan; shien-c@city.isesaki.lg.jp

**Keywords:** prevention of dementia, community-dwelling elderly, physical activity

## Abstract

In a rapidly aging society, like that in Japan, it is imperative to establish strategies to prevent dementia. We investigated the effects of a dementia prevention program, conducted as part of a municipality’s long-term care prevention project, on cognitive/motor functions among the community-dwelling elderly. Participants underwent a physical activity program involving muscle training and aerobic exercise, once a week, for 12 weeks. Cognitive and motor tests were performed twice, before and after the intervention. Thirty participants, aged 75.7 ± 6.7 years, were included in the analysis. Scores from the immediate word memory task were significantly increased (23.0 ± 7.8 vs. 25.7 ± 6.5) after the program. Scores from the Yamaguchi Kanji Symbol Substitution Test were also significantly increased (36.2 ± 13.1 vs. 40.1 ± 14.1). Time spent during the 5 m maximum walking speed test was significantly shorter after the program (3.4 ± 0.8 vs. 3.0 ± 0.7 s), whereas the daily walking distance increased significantly (1.9 ± 1.5 vs. 3.1 ± 2.2 km). Participants showed improvement in some aspects of their cognitive/motor function and walking distance per day. Therefore, this program can be regarded as a practical community-based healthcare activity.

## 1. Introduction

In a rapidly aging society, like that in Japan, the number of elderly with dementia is expected to increase annually [1]. Therefore, it is imperative to take some preventive measures against dementia because it is the second leading cause of a requirement for nursing care among the elderly [2].

The “Basic Guidelines for the Promotion of Community Healthcare Measures” issued by the Ministry of Health, Labour and Welfare of Japan (MHLW) recommends volunteer work led by community residents and in-group activities to promote community healthcare [3]. Municipalities should support these activities. However, it is challenging for municipalities to implement long-term care (LTC) prevention projects throughout the community, or for all community residents, because of the cost and limited number of healthcare professionals. Therefore, programs implemented in coordination with residential organizations, such as community associations and senior citizens’ clubs, are necessary [4].

In 2006, the LTC insurance system of Japan was revised to place more importance on the prevention of dementia, along with the establishment of LTC prevention projects targeting the community-dwelling elderly [5,6]. These projects are implemented by municipalities and use the basic checklist to screen the elderly to accurately and efficiently identify individuals with frailty, who may progress to a state requiring support or LTC. LTC prevention projects are broadly divided into primary prevention services, which target the healthy elderly, and secondary prevention services, which target those who may require support or LTC. The secondary prevention services include dementia prevention programs, as well as activities aimed to improve motor/oral functions and dietary habits. Each municipality should develop its own original programs based on the characteristics of the community and targeted individuals.

Recent epidemiological research has suggested that regular physical activity, which helps to prevent cognitive decline among the elderly, contributes to a decreased incidence of dementia [7,8,9,10,11]. Thus, to prevent dementia, it is necessary to establish programs to help the community-dwelling elderly to start physical activities before entering the clinical stage. Physical activities, such as walking and stretching, are easy for the elderly to practice.

The aims of this study were to investigate the effects of a dementia prevention program on the cognitive and motor functions of the community-dwelling elderly and to determine whether this program helps them develop exercise habits.

## 2. Methods

### 2.1. Participants

The Isesaki City Community General Support Center sent the basic checklist by mail to 38,018 individuals aged ≥65 years who had not been certified as requiring support or nursing care as of May 2014. A total of 26,584 completed the checklists, which were then collected (response rate, 69.9%). Based upon the responses received, 7198 elderly people were selected as requiring secondary prevention services. Among these individuals, 1680, living in the three districts in which dementia prevention programs were planned, received a brochure announcing the program by mail. Of the 32 individuals who wished to attend the program, 31 consented to participate in our study. All participants, except the one who withdrew from the study because of an injury, attended eight or more of the 12 program sessions. Consequently, a total of 30 elderly people were analyzed. The medical ethics committee of Gunma University approved this study (approval number: 26–20), and written informed consent was obtained from all participants.

### 2.2. Evaluation

Before the initiation of the dementia prevention program, the public health nurses gave participants a self-completed questionnaire that inquired about the following items: subjective health status (1: very healthy; 2: moderately healthy; 3: not very healthy; and 4: not healthy), walking habits (walking distance and time per day), frequency of forgetfulness (1: never; 2: seldom; 3: sometimes; 4: often; and 5: always), degree of forgetfulness (1: very mild; 2: relatively mild; 3: appropriate for age; 4: relatively severe; and 5: very severe), and functional activities, which were assessed using the Tokyo Metropolitan Institute of Gerontology Index of Competence (TMIG-IC) [12]. The subjective health status and frequency and degree of forgetfulness were included to examine how they can be changed by the dementia prevention program. Participants completed the questionnaire and submitted it at the time of the baseline evaluation.

During the baseline evaluation, public health nurses assessed the participants’ general cognitive function using the Mini Mental State Examination (MMSE) [13]. The other cognitive function tests used in this study are described in the Effectiveness Evaluation Instructions of the Nursing Care Prevention Manual [14], which was developed by MHLW. They include the immediate word memory task (a scale to assess the immediate recall ability), delayed word memory task (a scale to assess the delayed recall ability), Yamaguchi Kanji Symbol Substitution Test (YKSST) (a scale to assess the executive function) [15], and word recall test (a scale to assess the language ability).

A 5 m maximum walking speed test and timed up-and-go test (TUG) were performed to evaluate the physical function of each subject. Improvement is reflected by a decrease in the time required to complete the 5 m maximum walking speed test and TUG.

During the second evaluation after finishing the program, a questionnaire survey, cognitive function tests other than MMSE, and physical function tests were performed.

### 2.3. Intervention

The dementia prevention program was implemented at a civic gymnasium once a week for 12 weeks. The mean duration of the program was 90 min. The physical activity program was the primary content of the program. The program included two types of muscle training exercises in a sitting position, two types of muscle training exercises in a standing position, and aerobic exercise. All these activities were performed under the direction of public health nurses. Participants were encouraged to perform the same activities, including walking, at their home daily. Pedometers were provided to the participants to record the number of steps taken each day.

The staff of the dementia prevention program comprised six public health nurses from the Isesaki City Community General Support Center, one nurse, and one physical therapist. In August 2014, a volunteer training workshop was held for dementia supporters (trained and certified volunteers who help patients with dementia and their family caregivers) living in the city. Among the 30 workshop attendants, 19 (68.8 ± 5.5 years; one male and 18 females) desired to cooperate in the dementia prevention program. Three to seven dementia supporters attended each program session to support the participants and assist the professional staff.

The program was conducted on the basis of the following five principles of brain-activating rehabilitation: (1) a comfortable atmosphere; (2) empathetic communication with each other; (3) praising each other; (4) having a social role; and (5) errorless learning [16]. The healthcare professionals and dementia supporters conducted the program in a manner that enabled the participants to feel the pleasure of participating, praise each other, actively communicate with each other, play different roles, and have a successful experience while trying not to make mistakes.

### 2.4. Statistical Analysis

We analyzed data from participants who had consented to cooperate in this study and participated in eight or more of the 12 program sessions, as well as both the baseline and second evaluations. To analyze the representative values obtained from the baseline and second evaluations, the paired *t*-test was used when the values showed a normal distribution and the Wilcoxon signed-ranks test was used when the distribution was not normal. These analyses were performed using the Japanese version of SPSS version 21 (IBM, Armonk, NY, USA). Differences resulting in *p* < 0.05 were regarded as significant. The effect size and power for the significant differences were calculated using G*power (Cognitive and Engineering Psychology, Heinrich Heine University, Düsseldorf, Germany).

## 3. Results

The participants comprised eight men (26.7%) and 22 women (73.3%), with a mean age of 75.7 ± 6.7 years. The average rate of attending the 12 program sessions was 92.1%. The average MMSE score was 27.7 ± 1.8 (range, 23 to 30).

Table 1 shows the results of the evaluations. Among the cognitive function tests, the scores of the immediate word memory task significantly increased after the program (23.0 ± 7.8 vs. 25.7 ± 6.5 points, *p* = 0.004, effect size: 0.373, power: 0.516). The YKSST scores also increased significantly (36.2 ± 13.1 vs. 40.1 ± 14.1, *p* = 0.009, effect size: 0.286, power: 0.329). The word recall test scores changed from 9.7 ± 2.7 to 10.7 ± 3.3, before and after the program, although the difference did not reach statistical significance (*p* = 0.104). There were no significant differences in the scores of the delayed word memory task before and after the program.

The time required to complete the 5 m maximum walking speed test was significantly shorter after the program (3.4 ± 0.8 vs. 3.0 ± 0.7 s, *p* = 0.003, effect size: 0.530, power: 0.801), suggesting an increase in the participants’ walking speed. The number of steps taken during the 5 m maximum walking speed test did not change significantly. The time required to complete the TUG test changed from 7.9 ± 2.1 to 7.4 ± 1.4 s, before and after the program; however, the difference was marginal (*p* = 0.081).

The participants’ daily walking distance became significantly longer after the program (1.9 ± 1.5 vs. 3.1 ± 2.2 km, *p* = 0.616, effect size: 0.530, power: 0.889). No significant differences were observed in the scores of the subjective health status, frequency of forgetfulness, degree of forgetfulness, TMIG-IC, or self-reported walking time per day.

## 4. Discussion

Here participants showed improvement in their cognitive and physical functions along with an increased walking distance in daily life. The average MMSE score of the participants suggests that they had relatively normal cognitive function. Among the elderly without cognitive impairment, aerobic exercise programs have beneficial effects on motor function, cognitive speed, auditory and visual attention [17], and executive function [18]. In addition, the programs reported to improve the cognitive function have a greater effect when combined with muscle training exercises [18]. The findings in these studies are consistent with the increased YKSST scores (a scale to assess the executive function in the elderly) shown by our participants who attended the 12-week physical activity class involving aerobic exercise and muscle training.

Executive function has been suggested to correlate with the ability to perform the activities of daily living (ADL) [19] and instrumental ADL (IADL) among the healthy elderly [20]. Therefore, this function can be regarded as an important outcome measure in dementia prevention programs designed to help the community-dwelling elderly lead an independent life. Here participants showed an improvement in the executive function, suggesting that the dementia prevention program prevents the cognitive decline that precedes the onset of dementia [21].

Cognitive frailty is proposed as a new clinical construct with coexisting physical frailty and cognitive impairment in nondemented older adults [22]. Reversible cognitive frailty is indicated by subjective cognitive decline (SCD), which is characterized by increasing compensatory cognitive efforts (normal cognitive performance range) and subtle cognitive decline, including subjective memory dysfunction [23]. The intervention program of the present study was designed as a primary prevention of dementia for the older people who are healthy and functionally independent at the present. The participants consisted of older people with and without subjective memory dysfunction. SCD can be an effective target for cognitive impairment intervention [24]. It is necessary to design cognitive interventions targeting SCD in future studies.

Regular exercise is, to a certain extent, effective in inhibiting the onset of dementia [9,11]. The subjects showed an improvement in walking speed, which indicates improvements in physical fitness. The increased daily walking distance observed in this study suggests that the dementia prevention program helps individuals increase their daily walking distance and provides them with opportunities to develop exercise habits in daily life. Increased walking distance may also reflect an improvement in endurance among the participants.

The beneficial effects of non-pharmacological therapy, including exercise programs, are effective based on the manner in which they are provided, rather than their content [16]. Therefore, in the present study, the exercise class was developed on the basis of the five principles of brain-activating rehabilitation and held in a manner to enable participants to perform physical activities while enjoying interaction with other participants. The dementia supporters who joined the present intervention enabled smooth implementation of the program. In addition, they facilitated a pleasant atmosphere and easy communication among participants. Municipalities are required to tackle community healthcare issues in coordination with community-dwelling volunteers [3]. Because the dementia prevention program conducted in this study was a part of the Isesaki City’s Long-Term Care Prevention Program to which the dementia supporters contributed it can be used as a practical community-based healthcare activity.

However, the present study has several limitations. The study participants desired to attend the program; therefore, these individuals may be more health-conscious than others. Changes in functional activities among the participants could not be accurately evaluated because of the ceiling effect of TMIG-IC. The number of participants involved was small (*n* = 30), and the study period was short (12 weeks). A longer-term follow-up of the participants should be conducted to evaluate whether the positive effects are long-lasting. The present intervention study involved only a single group. It is necessary to conduct a controlled trial to investigate the effects of the program. The physical activity programs implemented in this study have improved the immediate word memory task and YKSST scores. However, the scores of the delayed word memory task and word recall test did not improve. It is necessary to clarify how the physical activity programs positively impacts the cognitive function domain. Although the executive function of the participants improved, its influences on ADL and IADL was not evaluated. Instead, we measured TMIG-IC because dementia prevention programs aim to maintain the independence of the community-dwelling elderly. Therefore, it is necessary to include the outcome measures of the abilities of participants to perform ADL and IADL in future studies.

## 5. Conclusions

Participants in a municipality-implemented dementia prevention program based on exercise and supported by local dementia supporters showed improvement in some aspects of their cognitive and physical functions. Thus, the program can be regarded as a practical community-based healthcare activity and a primary dementia prevention program based on a population strategy.

## Figures and Tables

**Table 1:** Results of the test scores (*n* = 30).

Scale	Baseline Evaluation ^†^	Second Evaluation ^†^	*p*-Value
Immediate word memory task	23.0 ± 7.8	25.7 ± 6.5	0.004 ** ^a^
Delayed word memory task	5.8 ± 3.5	6.4 ± 3.1	0.328 ^a^
YKSST	36.2 ± 13.1	40.1 ± 14.1	0.009 ** ^a^
Word recall test	9.7 ± 2.7	10.7 ± 3.3	0.104 ^a^
Subjective health status	2.4 ± 0.6	2.4 ± 0.6	0.739 ^b^
Frequency of forgetfulness	3.4 ± 0.6	3.3 ± 0.7	0.257 ^b^
Degree of forgetfulness	3.1 ± 0.8	3.1 ± 0.8	0.564 ^b^
Self-reported walking distance per day (km)	1.9 ± 1.5	3.1 ± 2.2	0.020 * ^b^
Self-reported walking time per day (min)	37.0 ± 23.0	48.1 ± 33.1	0.113 ^b^
TMIG-IC	11.8 ± 1.9	11.9 ± 2.1	0.593 ^b^
TUG	7.9 ± 2.1	7.4 ± 1.4	0.081 ^a^
5 m maximum walking speed test: time (s)	3.4 ± 0.8	3.0 ± 0.7	0.003 ** ^a^
5 m maximum walking speed test: steps	8.3 ± 2.1	7.8 ± 1.3	0.133 ^a^

**^†^** Results are expressed as the mean ± standard deviation. YKSST, Yamaguchi Kanji Symbol Substitution Test; TMIG-IC, Tokyo Metropolitan Institute of Gerontology Index of Competence; TUG, timed up-and-go test. ^a^ Paired *t*-test; ^b^ Wilcoxon signed-ranks test; * *p* < 0.05; ** *p* < 0.01.
